# Effectiveness of auriculotherapy on anxiety during labor: a randomized clinical trial^1^

**DOI:** 10.1590/1518-8345.2471.3030

**Published:** 2018-09-06

**Authors:** Reginaldo Roque Mafetoni, Mariana Haddad Rodrigues, Lia Maristela da Silva Jacob, Antonieta Keiko Kakuda Shimo

**Affiliations:** 2 PhD, Adjunct Professor, Instituto de Ciências da Saúde, Universidade Paulista, Campinas, SP, Brazil.; 3 PhD, RN, Secretaria Municipal de Saúde, Prefeitura Municipal de Sorocaba, Sorocaba, SP, Brazil.; 4 Doctoral student, Faculdade de Enfermagem, Universidade Estadual de Campinas, Campinas, SP, Brazil. Assistant Professor, Faculdade de Jaguariúna, Centro Universitário de Jaguariúna, Jaguariúna, SP, Brazil.; Centro Universitário de Jaguariúna, Jaguariúna, SP, Brazil; 5 PhD, Associate Professor, Faculdade de Enfermagem, Universidade Estadual de Campinas, Campinas, SP, Brazil.

**Keywords:** Auriculotherapy, Acupuncture, Ear, Complementary Therapies, Labor, Obstetric, Obstetric Nursing, Anxiety

## Abstract

**Objective:**

to evaluate the effectiveness of auriculotherapy on the anxiety of women during labor.

**Method:**

this is a randomized, parallel, triple-blind clinical trial. 102 parturients with gestational age ≥ 37 weeks, cervical dilatation ≥ 4 cm and two or more contractions in 10 min were selected and randomly assigned into three groups to receive auriculotherapy, placebo or control (routine care). Auriculotherapy was applied with crystal microspheres to the *shenmen*, *uterus*, *neurasthenia area* and *endocrine* points, and anxiety was assessed by the Hamilton Anxiety Rating Scale (HAM-A). Analyzes were performed using the Kruskal-Wallis, Generalized estimating equations, Chi-square and Fisher’s exact tests.

**Results:**

the groups showed no significant difference at baseline according to the HAM-A. After the intervention there was a significant increase in HAM-A scores at 120 min in the placebo versus auriculotherapy group (mean difference (MD) 3.62, confidence interval (CI) 0.42-6.81, p=0.0265) and control versus auriculotherapy group (MD 4.88, CI 1.87-7.88, p=0.0015).

**Conclusion:**

the parturients with auriculotherapy presented lower levels of anxiety according to the HAM-A score after the treatment when compared to the women from the other groups; this can represent alternative care in obstetric practice. Registration: n. RBR-47hhbj.

## Introduction

Anxiety is a common symptom faced by women during labor which is often related to a lack of information about gestation and delivery during prenatal care, or may arise from a new and unknown situation such as birth to first-time mothers^1^. Anxiety disorders and distress experienced by women during gestation may also be related to postpartum depression, premature birth, cesarean delivery and difficulties in raising the children^2^. However, there is evidence that prenatal education and guidance on physiological changes during gestation and the labor and delivery process may result in less stress, female anxiety and reduce interventions during childbirth^1^ ^,^ ^3^ ^-^ ^4^.

Anxiety brings a perception of harm or threat that can produce feelings of worry and fear concerning the possibility of physical or psychological damage. It is accompanied by physical changes and similar behaviors, which may also cause fear^5^. Thus, anxiety is a combination of the emotions of worry and fear. For Traditional Chinese Medicine (TCM), anxiety can be explained by a deficiency of substances called *xue* or *yin* (energy that produces cold), or by disharmonious patterns of excess heat energies (*yan* energy), or even both at the same time^6^. 

There are currently some Complementary and Integrative Health Practices (CIHP) used to relieve pain and anxiety during labor^7^ ^-^ ^8^. Such therapies are conducted in a less invasive and low-cost manner, which can increase comfort and promote the physiological evolution of the labor process; however some bias in the studies limits their use in the practice. 

Auriculotherapy or auricular acupuncture is a TCM modality that uses reflex points in the ear to the central nervous system to treat various disorders of the body through stimulation with needles, pressure with seeds or microspheres^9^. 

 Stimulation of auriculotherapy points for treating anxiety has been evaluated by some studies. One study^10^ performed with health professionals associated the use of this therapy with a significant reduction in anxiety, burnout and traumatic stress, and observed a significant increase in professional courage and patience. Another study^11^ showed a reduction of 20.97% in the anxiety level of nursing students after stimulus with semipermanent needles in the *shenmen* and *brainstem* ear points. 

The Chinese auricular map describes some points to treat anxiety^11^ ^-^ ^12^, obstetric dystocias, labor induction and (labor) pain^13^, which may represent complementary care during labor and delivery. However, the lack of evidence and auriculotherapy studies in women during labor limits professionals’ knowledge and the establishment of this therapy in obstetric practice. In this context, the present study aimed to evaluate the effectiveness of auriculotherapy on women’s anxiety level during labor.

## Method

This is a parallel, pragmatic, randomized clinical trial (RCT)^14^ conducted from April 2015 to June 2016, in which 102 parturients admitted to a public university hospital in the State of São Paulo, Brazil participated. Pregnant women of any age or parity after 37 weeks of gestation, in spontaneous, induced and/or accelerated labor, with two or more contractions in 10 minutes, dilation ≥ 4 cm, undamaged ear skin, and a living fetus with good vitality were included in the study. Cases of dilatation ≥ 7 cm, severe pre-eclampsia, previous cesarean section, immediate indication of cesarean section, or use of analgesic medication less than six hours from admission were excluded from the study.

The sample size was estimated considering the proposed method by an ANOVA model since these variables are quantitative and three groups were compared in this study. Estimates of the variable means for each of the groups and the standard deviation of the model’s mean square error were based on two previous studies^15^ ^-^ ^16^ which evaluated the effects of acumpuncture on the *Sanyinjiao* pressure point on labor pain and duration. A significance level of 5% and a test power of 80% were adopted for the calculations. The calculation resulted in a sample of 17 individuals per group for the pain intensity variable, and a sample of 33 individuals per group for the labor duration variable. It should be considered that this RCT is part of a larger study which evaluated other parameters of interest. The calculation resulted in a total sample of 99 individuals, which was the highest value found.

The allocation sequence was defined for 102 participants, being three more than the sample calculation for predicting losses, using sealed and numbered opaque envelopes, sequentially generated by the site *http:/www.randomization.com* by a statistics professional, who was not a member of the study. They were opened after including the parturient in the study by the principal investigator in the presence of two staff members of the unit, who were not participants of the assessment team or involved in direct care to the study’s parturients. Three groups were allocated: an intervention group (IG) with 1.5 mm polished crystal microspheres; a placebo group (PG) with glass microspheres, similar to the crystal ones; and a control group (CG) without intervention. The study used the triple-blind method; in this sense, individuals from the IG, the PG, the evaluators, the professionals who provided care in the obstetric unit and the statistics professional did not know which group any participant belonged to. It was not possible to blind participants, evaluators, or professionals regarding the CG due to the characteristics of the study.

The Hamilton Anxiety Rating Scale was used (HAM-A, 1959)^17^ to assess the anxiety level of the parturients, which comprises 14 items distributed into two groups. The first group has seven items related to anxious mood symptoms, while the second group also has seven items, but are related to physical symptoms of anxiety. Among the mood scales used worldwide, translated and adapted to the Brazilian reality regarding anxiety, the Hamilton’s Anxiety Scale presents easy applicability and reliability^18^. Although there are other scales for assessing anxiety, many of them were constructed as modifications of Hamilton’s proposed instrument. Since its publication (1959), the HAM-A has been used worldwide in several studies with clinical and academic purposes^19^. The total score is obtained by a sum of the values (degrees) assigned to all 14 items on the scale. The anxiety levels according to the HAM-A are: None = 0; Mild = 1; Moderate = 2; Severe = 3; Very Severe = 4. The sum of the scores obtained on each item results in a total score ranging from 0 to 56. This score is classified according to the following intervals: 0 (zero) absence of anxiety; 1 (one) to 17 points, mild anxiety; 18 to 24 points, moderate anxiety; and 25 to 56 points, severe or intense anxiety. 

For the sociodemographic and clinical data collection, an instrument was developed and submitted to a content validity analysis performed by five judges with experience in obstetrics and/or TCM. 

The evaluation team was formed by five staff members of the obstetric nursing team unit who were instructed about the objectives of this study, previously trained for data collection and the application of the HAM-A upon study admission and at 120 minutes of treatment. The parturients were approached in the preterm delivery rooms of the obstetric center, assessed for eligibility, invited to participate in the study, then later allocated by drawn order and accompanied by the team of evaluators until the day after delivery when the microspheres were removed. The principal investigator had taken two technical short-courses training sessions totaling 64 hours, and was responsible for applying the auriculotherapy to the IG and PG (sham points).

**Figure 1:**
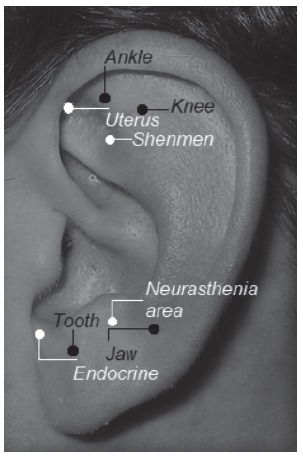
Location of the ear pressure points used in the study. Campinas, SP, Brazil, 2016

The following auriculotherapy points were used in the IG: i) *shenmen*, which predisposes the brainstem and the cortex to receive, condition and encode auricular reflexes, with sedatives and analgesic effects; ii) *uterus*, which is indicated for gynecological and obstetric changes, labor induction or reduction of the expulsion period and to reduce postpartum pain; iii) *neurasthenia area*, indicated for treating anxiety; and iv) *endocrine*, which regulates the functions of endogenous secretory glands, used in gynecological disorders^11^ ^-^ ^13^. Sham points were used in the PG (which are not indicated for the proposed treatment): *ankle, knee, tooth and jaw*, as shown in Figure 1. 

The ear lobe was previously cleaned with 70% ethyl alcohol, and the points were searched for by pressure exerted by the Point Probe Tool/point finder and defined close to the topography indicated on the ‘point map’ as those more painful to palpation. Once the point was located, the crystal sphere was attached with adhesive tape to the IG participants and individually pressed for one minute or until causing tolerable pain to induce the stimulus. For the PG, the Point Probe Tool/point finder was used to indicate the location and place the glass microspheres on the *sham* points. Parturients allocated in the CG were followed up for the same period and evaluated by HAM-A as in the other groups. 

Preparation and previous instructions about labor evolution are carried out during prenatal consultations or in specific courses offered by the municipal health network. Some Complementary and Integrative Health Practices (CIHP) are available at the study site and are targeted and offered to parturients, such as: shower, lombossacral massage, breathing exercises, and the Swiss/exercise ball; and they have freedom of movement in cases without medical contraindication. The study site also allows a companion of the woman’s choice during labor and delivery, as well as accomodation in joint housing. However, induced and accelerated labor by intravenous oxytocin, prostaglandins and amniotomy are routinely used.

Comparisons between the groups regarding the quantitative variables were performed using the Kruskal-Wallis test. This test is a non-parametric test, similar to the ANOVA model. Comparisons between groups and evaluation periods were performed using *Generalized estimating equations* - GEE models. The mean difference estimates were presented, as well as their respective confidence intervals and p-values. The Chi-square test or Fisher’s exact test were used for the associations between the groups and the categorical variables. The analyzes were performed by a statistician using the *Statistical Analysis System* (SAS) 9.4 software.

The study project was submitted to the evaluation and approval by the Local Research Ethics Committee (Opinion number 855.496). The parturients who accepted to participate in the study signed a clear and informed consent form, in compliance with the legislation in force in the country.

## Results

**Figure 2:**
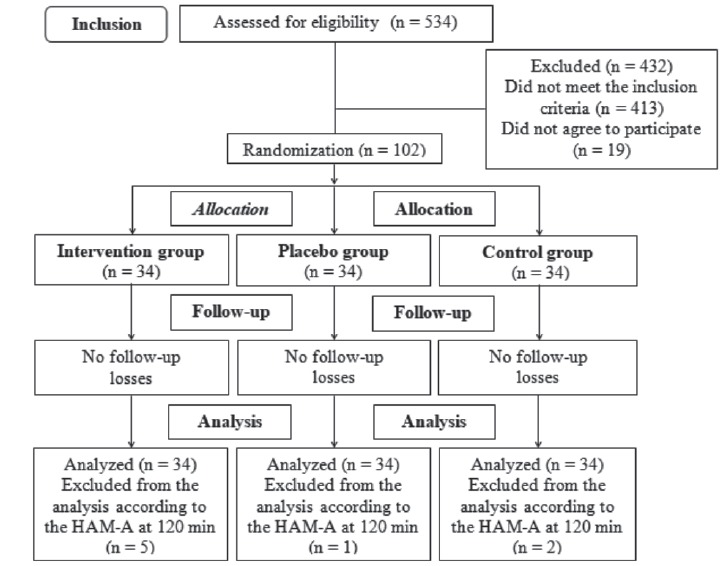
Flow diagram of the recruitment and grouping of the participants. Campinas, SP, Brazil, 2016

A total of 102 parturients (three groups of 34) participated in this study. The 120-min HAM-A reassessment analyzes of parturients whose labor occurred before this period were excluded (Figure 2). Data from the total sample was considered (102 parturients, 34 per group) for the other variables and parameters of interest of the study.

No significant difference were observed among the three groups for the variables: mean age of the parturients (23.9 (± 5.8) in the IG, 25.1 (± 7.0) in the PG and 22.7 (± 5.3) years in the CG, p-value = 0.3503, Kruskal-Wallis test); and marital status, in which the majority lived with a companion (IG: 29 (85.3%) *versus* PG: 33 (97.1%) *versus* CG: 32 (94.1%), p-value = 0.2674, Fisher’s exact test). Having a companion of choice during hospitalization was frequent in all three groups (IG: 33 (97.1%) *versus* PG: 31 (91.2%) *versus* CG: 32 (94.1%), p-value=0.7613) and sharing the room with other parturients, which is routine in the unit, was similar among the groups (IG: 13 (38.3%) *versus* PG: 15 (44.1%) *versus* CG: 12 (35.3%), p-value=0.7498). 

The variables schooling, previous guidance on labor and use of CIHPs (including auriculotherapy) sought to assess the degree of general and specific instruction of the woman, which could influence their behavior during labor. The schooling level was measured in study years (IG: 10.7 (± 3.1); PG: 9.9 (± 2.4); CG: 10.7 (± 3.1); p-value=0.4567). The majority of parturients did not participate in preparation courses, nor did they receive guidance on the labor process or use of CIHPs during the delivery process (IG: 29 (85.3%); PG: 30 (88.2%) and; CG: 29 (85.3%), p-value=1.000). A lack of knowledge about auriculotherapy was also similar in the sample, described as “I have never heard of it” by 22 (64.7%) of the women in the IG, 20 (58.8%) in the PG, and 19 (55.9%) in the CG, p-value = 0.7375. 

Upon the study admission, a greater number of women in spontaneous labor was observed in the IG (IG: 25 (73.5%) *versus* PG: 16 (47.1%) *versus* CG: 09 (55.9%), p-value=0.0781, Chi-square test), although not statistically significant. No significant differences were observed comparing the three study groups regarding accelerated labor with medications and other obstetric measures listed in Table 1, which may represent the initial first stage of labor or the advanced stage.

**Table 1:** Distribution of parturients according to study groups and obstetric characteristics. Campinas, SP, Brazil, 2016

Variable	Study group			
	Intervention (N = 34)	Placebo (N = 34)	Control (N = 34)	p-value
No. of prenatal consultations, mean (SD)*	9.3 (2.2)	9.9 (2.4)	8.9 (2.5)	0.4492^†^
No. of gestations, mean (SD)*	1.6 (1.0)	1.6 (1.0)	1.7 (1.1)	0.8193^†^
Parity, n (%)				
Nulliparous	27 (79.4)	25 (73.5)	25 (73.5)	0.8090^‡^
Multiparous	07 (20.6)	09 (26.5)	09 (26.5)	
Prior to the treatment				
Amniotic membranes, n (%)				
unruptured	25 (73.5)	21 (61.8)	19 (55.9)	0.3378^§^
ruptured	09 (26.5)	13 (38.2)	15 (44.1)	
Cervical dilation (cm), mean (SD)*	4.6 (0.9)	4.8 (0.8)	4.5 (0.8)	0.3915^†^
No. of contractions, mean (SD)*	3.1 (0.9)	3.2 (0.7)	3.3 (0.9)	0.5986^†^
Intensity of contractions, n (%)				
Mild	2 (5.8)	6 (17.6)	0 (0.0)	
Moderate	16 (47.1)	17 (50.0)	22 (64.7)	0.0634^§^
Severe	16 (47.1)	11 (32.4)	12 (35.3)	
After treatment				
Accelerated labor	n (%)	n (%)	n (%)	
Prostaglandin	07 (20.6)	10 (29.4)	11 (32.4)	0.5273^‡^
Oxytocin	18 (52.9)	16 (47.1)	21 (61.8)	0.4725^‡^

p < 0.05. *standard deviation ^†^Kruskal-Wallis test ^‡^Chi-square test ^§^Fisher’s exact test

Table 2 shows the means of the HAM-A scores and the categorized anxiety levels before and after treatment among the study groups.

**Table 2:** Differences in HAM-A* and degree of anxiety among study groups. Campinas, SP, Brazil, 2016

Variable	Study group		
	Intervention	Placebo	Control
	(N = 34)	(N = 34)	(N = 34)
Prior to treatment	mean ± SD^†^	mean ± SD^†^	mean ± SD^†^
HAM-A*	5.6 ± 4.5	5.6 ± 5.5	6.8 ± 5.7
Anxiety level	n (%)	n (%)	n (%)
None	00 (0.0)	04 (11.8)	04 (11.8)
Mild	33 (97.1)	29 (85.3)	28 (82.3)
Moderate	01 (2.9)	01 (2.9)	02 (5.9)
Severe	00 (0.0)	00 (0.0)	00 (0.0)
	(N = 29)	(N = 33)	(N = 32)
120 min after treatment	mean ± SD^†^	mean ± SD^†^	mean ± SD^†^
HAM-A*	5.7 ± 5.0	9.3 ± 7.9	10.5 ± 7.1
Anxiety level	n (%)	n (%)	n (%)
None	03 (10.3)	03 (9.1)	01 (3.1)
Mild	26 (89.7)	21 (63.6)	25 (78.1)
Moderate	00 (0.0)	09 (27.3)	04 (12.5)
Severe	00 (0.0)	0.0 (0.0)	02 (6.3)

*HAM-A - Hamilton Anxiety Rating Scale ^†^standard deviation

Table 3 shows the mean differences in HAM-A scores before and after 120 minutes of treatment. In analyzing the scores before and at 120 minutes among each group, a significant difference was found in the PG (MD 3.64, CI 2.26-5.02, p<0.0001) and CG (MD 3.71, CI 2.40-5.04, p<0.0001) due to the increase in the means, which did not occur in the IG (MD 0.07, CI -0.61-0.75, p=0.8429). 

**Table 3:** Comparisons in the HAM-A* scores and moments of evaluation among the study groups. Campinas, SP, Brazil, 2016

Comparison	Mean difference	Confidence interval (95%)		p-value
		Lower limit	Upper limit	
HAM-A*, prior to treatment				
Intervention *vs* placebo	0.05	-2.42	2.52	0.9682^†^
Intervention *vs* control	1.23	-1.31	3.77	0.3441^†^
Placebo *vs* control	-1.18	-3.88	1.53	0.3938^†^
HAM-A*, at 120 min of treatment				
Intervention *vs* placebo	3.62	0.42	6.81	0.0265^†^
Intervention *vs* control	4.88	1.87	7.88	0.0015^†^
Placebo *vs* control	-1.26	-4.84	2.33	0.4915^†^

*HAM-A - Hamilton Anxiety Rating Scale

p < 0.05. †GEE test - *Generalized estimating equations*

On the day after delivery, the parturients responded affirmatively when asked if they would undergo auriculotherapy again in a future pregnancy: 33 (97.1%) in the IG and 29 (85.3%) in the PG (p=0.1974). No difference was observed when they were asked about the discomfort caused by auriculotherapy (“It did not bother me”: 30 (88.2%) in the IG *versus* 31 (91.2%) in the PG; “It bothered me a little”: 4 (11.8%) in the IG *versus* 3 (8.8%) in the PG, p=1.0000, Fisher’s exact test).

## Discussion

Anxiety is a common symptom faced by women during childbirth; especially when it involves low-educated parturients, primigravidae women and hospital environments with high rates of medical interventions^20^. This study aimed to evaluate the effects of auriculotherapy as a CIHP on routine care support offered to parturients, which showed control of the anxiety in the IG according to the Hamilton anxiety rating scale^17^. 

The parturients in this study had similar previous education or preparation for childbirth when evaluating: schooling, the number of prenatal consultations and the low participation in courses on childbirth. In addition, nulliparity, the presence of a companion of their choice and a shared room with other parturients were similar among the groups and are important factors impacting the anxiety level and the satisfaction of women with their birthing expericence^21^. Some obstetric characteristics considered to cause pain and which can consequently increase the level of anxiety^22^ did not statistically differ between the groups; these included the intensity and number of contractions, and inducing or accelerating labor with prostaglandins or ocytoxin.

The birthing process accounts for more than 90% of a woman’s stress and anxiety during prenatal care, mainly related to lack of knowledge and fear of childbirth^23^. The presence of anxiety symptoms was characteristic in the PG (>88%), CG (>88%) and IG (100%) groups, which may be related to the low prenatal education observed in all three study groups (>85%). Regarding the HAM-A scores, the comparisons were statistically significant for all analyzes when comparing IG versus PG and IG versus CG 120 minutes after allocation in the study, which did not occur in the comparisons between PG and CG at the same time. This may have occurred due to the real stimulation of ear pressure points in the IG which was not applied in the PG and CG groups, with a later increase in HAM-A scores for these groups. 

The use of auriculotherapy during labor was also evaluated in a study with 80 parturients to compare the effects of this therapy on pain during the active phase of labor through a visual analogue scale (VAS)^13^. In their results, the mean pain intensity after treatment was significantly lower in the IG (IG: 7.56 versus CG: 8.43 p <0.05). The fact that this therapy alone favors pain relief already reduces the symptoms of anxiety^22^, which may have also occurred in this RCT in the IG after 120 minutes.

In a Cuban study^24^, researchers evaluated the use of auriculotherapy on the anxiety of women awaiting an abortion curettage. The sample consisted of 48 women (90.5%, n = 53) and the *shenmen*, *heart* and *anxiolytic* pressure points were stimulated with thistle seeds (*Argemone mexicana L.*), pressing the points three times a day over seven days prior to the day of the procedure. Unlike the present study in which women were classified with low anxiety level, Cuban women were admitted with a high degree of anxiety (scores ≥ 45 points) by the Spielberger’s State-Trait Anxiety Inventory (STAI). Thus, the results were clearer after treatment; only three women (5.66%) presented low anxiety (-30 points), one (1.88%) presented moderate anxiety (3044 points), while the others did not present anxiety symptoms. Auriculotherapy was effective in reducing the anxiety symptoms of women who needed to end gestation/go into labor, and they also did not increase the anxiety symptoms of women who received this therapy during the labor process (IG), as shown in our results. 

Regarding this subject, studies on reflexology^8^ ^)^ and aromatherapy^25^, and (those) restricted to auriculotepia studies for these characteristics were found in the literature as an alternative treatment for controlling women’s anxiety during labor. Reflexology showed a significant reduction in the anxiety level according to the STAI (p<0.001) after treatment when compared to those women under routine care^8^. In a study on aromatherapy (use of lavender essential oil) with 121 nulliparous women, a reduction in STAI scores (p<0.05) and cortisol concentration was also observed, while plasma concentrations of serotonin and 5-hydroxyl indole acetic acid (5-HIAA) increased, thereby improving labor progress^25^. Auriculotherapy in the obstetric specialty was found in an RCT^26^ with 76 postpartum women after cesarean section. This study showed a significant reduction in cortisol levels (mean difference (MD) = 4μg / dl, p <0.05), heart rate (MD = 9.2 beats/min, p <0.001) and anxiety (STAI) (MD = 3.8, p <0.01) after auriculotherapy (*shenmen* point).

A review study^12^ evaluated the effects of acupuncture in the treatment of anxiety; three RCTs included showed a reduction in the anxiety level after ear acupuncture in different scenarios than those of the present RCT (during pre-hospilatar transport, preoperatively and with healthy volunteers). The authors of this review described ear acupuncture as promising for incorporating anxiety treatment into clinical nursing practice, as well as an alternative that may reduce drug use for anxiety^12^. Acupuncture in Brazil is a nursing specialization that is legitimized by resolution no. 326 of 2008 of the Federal Nursing Council (COFEN)^27^, carried out in public and private health services for treating several symptoms; other health professionals with higher education and specialization courses also perform the technique. Health Complementary and Integrative Health Practices (CIHP) have been recommended by the World Health Organization (WHO) to be used in health systems around the world in order to broaden the therapeutic options and complement conventional treatments^28^. 

We can highlight the absence of data on diagnosed cases of anxiety disorders or previous and routine uses of medications for treating anxiety as a limitation of this RCT, although the sample was randomized and the anxiety symptoms were measured by the HAM-A upon study admission. Another limitation is the lack of data on parturients who used any type of routine CIHP in the obstetric unit, as these activities do not require adhesion and there is great variation regarding their frequency and usage period during labor.

## Conclusion

In this study, women undergoing stimulation by crystal microspheres on the following ear pressure points: *shenmen, uterus, neurasthenia area* and *endocrine*, maintained control of their anxiety levels during the active phase of labor when there is generally an increase in anxiety, as evidenced in the PG and CG groups without this same treatment; the fact that it did not increase the anxiety scores in the IG already indicates a useful effect of this therapy. However, this study coupled with new RCTs for auriculotherapy to assess parturient anxiety and conducted in less interventional environments will contribute with greater evidence for the establishment of this therapy in obstetric care.
